# Evaluation of the effectiveness of an incentive strategy on the questionnaire response rate in parents of premature babies: a randomised controlled Study Within A Trial (SWAT) nested within SIFT

**DOI:** 10.1186/s13063-021-05515-y

**Published:** 2021-08-21

**Authors:** Edmund Juszczak, Oliver Hewer, Christopher Partlett, Madeleine Hurd, Vasha Bari, Ursula Bowler, Louise Linsell, Jon Dorling, on behalf of the SIFT Investigator Group, Vasha Bari, Janet Berrington, Ursula Bowler, Elaine Boyle, Jon Dorling, Nicholas Embleton, Oliver Hewer, Madeleine Hurd, Samantha Johnson, Edmund Juszczak, Andrew King, Alison Leaf, Louise Linsell, Kenny McCormick, William McGuire, David Murray, Christopher Partlett, Tracy Roberts, Ben Stenson

**Affiliations:** 1 National Perinatal Epidemiology Unit Clinical Trials Unit, Nuffield Department of Population Health, University of Oxford, Oxford, UK; 2 Nottingham Clinical Trials Unit, School of Medicine, University of Nottingham, Nottingham, UK; 3 Division of Neonatal-Perinatal Medicine, Dalhousie University, Halifax, Canada

**Keywords:** Incentive, Effective, Response, Unconditional, Questionnaire

## Abstract

**Background:**

Loss to follow-up resulting in missing outcomes compromises the validity of trial results by reducing statistical power, negatively affecting generalisability and undermining assumptions made at analysis, leading to potentially biased and misleading results. Evidence that incentives are effective at improving response rates exists, but there is little evidence regarding the best approach, especially in the field of perinatal medicine. The NIHR-funded SIFT trial follow-up of infants at 2 years of age provided an ideal opportunity to address this remaining uncertainty.

**Methods:**

Participants: parents of infants from participating neonatal units in the UK and Ireland followed up for SIFT (multicentre RCT investigating two speeds of feeding in babies with gestational age at birth < 32 weeks and/or birthweight < 1500 g). Interventions: parents were randomly allocated to receive incentives (£15 gift voucher) before or after questionnaire return. The objective was to establish whether offering an unconditional incentive in advance or promising an incentive on completion of a questionnaire (conditional) improved the response rate in parents of premature babies. The primary outcome was questionnaire response rate. Permuted block randomisation was performed (variable size blocks), stratified by SIFT allocation (slower/faster feeds) and single/multiple birth. Multiple births were given the same incentives allocation. Parents were unaware that they were in an incentives SWAT; SIFT office staff were not blinded to allocation.

**Results:**

Parents of 923 infants were randomised: 459 infants allocated to receive incentive before, 464 infants allocated to receive incentive after; analysis was by intention to treat. Allocation to the incentive before completion led to a significantly higher response rate, 83.0% (381/459) compared to the after-completion group, 76.1% (353/464); adjusted absolute difference of 6.8% (95% confidence interval 1.6% to 12.0%). Giving an incentive in advance is the more costly approach, but the mean difference of ~£3 per infant is small given the higher return.

**Conclusions:**

An unconditional incentive in advance led to a significantly higher response rate compared to the promise of an incentive on completion. Against a backdrop of falling response rates to questionnaires, incentives can be an effective way to increase returns.

**Trial registration:**

SIFT (ISRCTN76463425). Registered on March 5, 2013.; SWAT registration (SWAT 69 available from http://www.qub.ac.uk/sites/TheNorthernIrelandNetworkforTrialsMethodologyResearch/FileStore/Filetoupload,864297,en.pdf). Registered on June 27, 2016.

**Supplementary Information:**

The online version contains supplementary material available at 10.1186/s13063-021-05515-y.

## Background

Missing data due to loss to follow-up has a widely detrimental effect on research studies. It can compromise internal and external validity of results for several reasons, including diminished statistical power due to a smaller effective sample size, a negative impact on generalisability, and the potential to bias results [1]. The implementation of strategies that effectively retain participants is therefore very important. The use of clinical assessments for follow-up can be very costly, so perinatal trials often use bespoke research questionnaires to collect data, including the primary outcome. It is therefore essential to mitigate loss to follow-up by maximising the return rate of questionnaires.

Systematic reviews suggest that incentives are effective at improving response rates for research questionnaires in clinical trials [2, 3]. Brueton [2] and Khadjesari [4] both reported that offering a monetary incentive improved questionnaire return rates compared with no incentive, in postal and online settings respectively. Edwards [3] reported that unconditional incentives (i.e. a reward given in advance as a goodwill gesture) led to superior response rates compared with conditional incentives (i.e. the promise of a reward on receipt of a questionnaire). However, there was significant heterogeneity among these studies. Dillman [5] and Singer and Ye [6] also reported results that favoured unconditional over conditional incentives.

However, the use of financial incentives is costly, and studies may not have the funds or resources to provide unconditional incentives. A conditional incentive, promised on receipt of a completed questionnaire, could be a more cost-effective means of enhancing retention by rewarding only those participants who respond, minimising wastage from monetary incentives sent out to non-responding participants.

There is also little evidence of which incentive methods are most effective in the context of follow-up of participants (via parents) in perinatal randomised controlled trials (RCTs). There is heightened sensitivity around the population (parents of vulnerable infants) and often a lengthy period between recruitment and outcomes captured at follow-up. Hardy et al. [7] successfully carried out a study of incentives at 1-year follow-up and reported it to be the only known study of incentives in this population. Kenyon et al. [8] investigated the effects of incentives in a perinatal trial, but this was for follow-up when the children were 7 years old. More recently, Bradshaw et al. investigated the use of incentives for 2-year follow-up in a perinatal trial, but this trial population excluded extremely preterm infants [9].

It is therefore prudent to use opportunities to narrow this evidence gap by investigating which method of incentive provides the greatest return, particularly in perinatal RCTs.

## Methods

### Aim, design and setting of the SWAT

The main objective of this Study Within A Trial (SWAT; for the definition of a SWAT visit https://www.nihr.ac.uk/documents/studies-within-a-trial-swat/21512) was to establish whether, in parents of preterm babies, offering an unconditional incentive in advance (with the first mailing of a questionnaire) or promising a conditional incentive (in the first mailing) on completion of a questionnaire, improves response rate. This randomised controlled SWAT was nested within the Speed of Increasing milk Feeds Trial (SIFT), a multicentre RCT in neonatal units in the UK and Ireland caring for very preterm or very low birthweight (VLBW) infants. SIFT recruited 2804 infants, randomised 1:1 to either a slower (18 ml/kg) or faster (30 ml/kg) daily increase in milk volume until reaching full feeds, between 8 June 2013 and 30 June 2015. The primary outcome was survival without moderate or severe disability at 2 years of age (corrected for prematurity), assessed by a questionnaire sent directly to parents (principally mothers). Questionnaires were sent (to all parents where infant(s) vital status and address could be confirmed) by both post and as a link to an online submission form via email and text message where these contact details were available. The SWAT investigated whether an unconditional incentive (a monetary voucher given before completion of a questionnaire) was more effective than a conditional incentive (the promise of a monetary voucher on receipt of a completed questionnaire) and has been reported elsewhere [10]. For ‘Before’ and ‘After’ letters and reminders see Additional file 1.

### Participant characteristics

Eligible participants were parents of infants recruited to SIFT who were due to be sent a questionnaire at 2 years of age (corrected for prematurity) at the SWAT start date (1 Feb 2017). SIFT recruited in 55 centres in the UK and Republic of Ireland. Eligibility criteria for inclusion in SIFT have been described elsewhere [11]. Participants were traced for survival and to confirm current residence. Where these details remained unknown, parents were not contacted. In addition, parents who had withdrawn consent to the 2-year follow-up were excluded.

### Interventions and comparisons

Participants were allocated randomly to one of two groups:

- A (After): the first letter posted to parents included a promise of an incentive (£15 gift voucher redeemable at high street shops) *after* receipt of a completed form.
- B (Before): the first letter posted to parents enclosed the incentive (£15 gift voucher redeemable at high street shops) *before* the receipt of a completed form.

Participants were randomised with an allocation ratio 1:1 by permuted block randomisation (using variable block sizes of 2, 4, 6 and 8 allocated in proportion to elements of Pascal’s triangle i.e., a ratio of 1:3:3:1) and stratified by original SIFT allocation (faster/slower) and by singleton/multiple birth by the Trial Statistician at the NPEU CTU. The interventions were assigned to the participants immediately because they had all been enrolled to SIFT at that point, so there was no gap during which the sequence needed to be concealed. Infants from multiple births were allocated to the same incentive group. Vouchers were allocated per questionnaire, so parents of multiple births received a voucher for each infant. SIFT office staff at the NPEU Clinical Trials Unit were aware of allocation due to the nature of the interventions and the practicalities involved in sending out the letters and the vouchers.

The incentive was a £15 high street shop voucher (€15 for participants recruited in the Republic of Ireland), sent via post. Reminder letters in both groups mentioned the incentives. Letters to those in the group allocated ‘After’ reiterated the promise of an incentive; letters to those in the group allocated ‘Before’ tactfully mentioned the incentive sent with the first letter. Parents were also reminded to complete the questionnaire via text and/or email during the follow-up window. All parents were offered the option of completing the questionnaire online or, as a last resort, via telephone.

The incentives SWAT was implemented midway through the SIFT follow-up. Parents of SIFT participants who had returned their follow-up questionnaire prior to the incentives study being implemented were sent a £15 voucher in a spirit of fairness.

### Outcomes

The primary outcome was the occurrence of questionnaire return, defined as receipt of a completed or partially completed questionnaire at the SIFT office. A questionnaire was considered completed or partially completed if the first three out of the five subsections of the questionnaire were completed (as these sections were required for the derivation of the primary outcome for the main SIFT trial).

Secondary outcomes included:

- Primary method of completion (paper, online, telephone)
- Total cost
- Number of reminders

Total cost included postage, receipt of material via prepaid Freepost envelope, cost of envelopes, supplementary materials (for example, sticker sets sent with questionnaires for infants to play with), and value of gift vouchers. It did not include Freepost licence fee, printing, telephone calls, or trial staff time. All costs for participants were calculated in GBP. The cost of the €15 vouchers sent to participants in the Republic of Ireland was converted to GBP using the exchange rate (via xe.com) on 10 May 2017, the date of the invoice for these vouchers. Costs for these participants also included Air Mail postage fees.

### Sample size

SIFT completed recruitment on 30 June 2015, with 2804 infants randomised. Given an expected 5% mortality rate in the patient population, it was estimated that 1250 of these infants would survive until the projected start date of the SWAT (originally 1 December 2016). It was also estimated that about 10% of the parents of these infants would be lost to the follow-up phase, due to withdrawal of consent or to lack of information on contact details or infant survival status (always checked prior to engaging with parents). This would result in approximately 1100 infants eligible for the SWAT, giving 550 per group.

It was estimated that the response rate with no incentives would be approximately 66%, based on experience from the BOOST-II UK trial with a similar patient population and 2-year follow-up methods [12].

Based on the most applicable literature on studies investigating incentives, it was anticipated that the addition of an incentive would result in an absolute increase of 10% in the response rate [4, 8]. Five hundred and fifty infants per group would allow detection of an absolute difference in response rate of around 7% at 90% power and a two-sided 5% level of significance.

### Statistical analysis

Infants (i.e. parent responses) were analysed in the groups to which they were randomly assigned, comparing the outcome of all infants allocated to ‘Before’ with all those allocated to the ‘After’ group, regardless of deviation from the protocol.

Baseline demographic information was summarised by randomised group using frequency counts and percentages for categorical data, means and standard deviations for normally distributed continuous data, or medians with interquartile ranges for other continuous data.

Comparative analysis entailed calculating the absolute difference in the proportion responding with corresponding 95% confidence interval (CI), and the difference in mean cost (plus 95% CI). In addition, the cost in £s per 1% increase in response rate was calculated, factoring in administration costs such as the number of reminder letters, as well as the monetary value of the incentive. For other outcomes relating to the method of completion and reminder letters, a similar strategy was used based on the distributions/type of data collected.

The principal comparison was the incentivised ‘Before’ group versus the incentivised ‘After’.

Pre-specified subgroup analysis examined the consistency of the effect of the timing of the incentive for the original allocation in SIFT (slower versus faster) and singleton versus multiple births, using the statistical test of interaction. An exploratory subgroup analysis exploring regional (England, Scotland, Wales, Northern Ireland, and Republic of Ireland) variation was also performed.

Pre-specified exploratory analysis examined the response rate in the period prior to the incentives study starting and during, overall (i.e. irrespective of incentive group allocation) with a 95% confidence interval.

No adjustment was planned for multiple testing since this SWAT involves a very small number of focused hypothesis tests.

## Results

### Participant flow and baseline characteristics

**Fig. 1:**
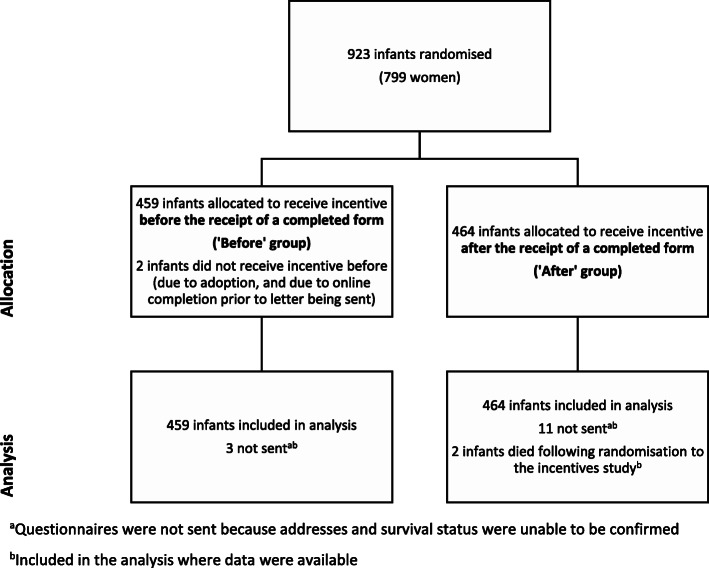
Flow of participants through the incentives SWAT

The incentives study began on 1 Feb 2017 and the last questionnaire received in the SIFT Office before data lock was on 12 March 2018. Nine hundred and twenty-three (923) infants were randomised to the SWAT (799 women). Four hundred and fifty-nine (459) of the infants were allocated to receive the incentive before completion (‘Before’ group). For this allocation, three questionnaires were not sent because addresses and survival status were unable to be confirmed. Four hundred and sixty-four (464) of the infants were allocated to receive the incentive after completion (‘After’ group). For this allocation, 11 were not sent due to unconfirmed address and survival status, and two infants were randomised in error; in both cases, the infants were later found to have died after they were randomised to the SWAT. All 923 infants were included in analysis (see Fig. 1). The two groups were well balanced across infant and maternal characteristics at trial entry (see Table 1).

**Table 1:** Infant and maternal characteristics at original SIFT trial entry

	Before group (***n*** = 459)	After group (***n*** = 464)
Number of centres, *n*	51	51
Allocated to faster group in SIFT ^a^, *n*/*N* (%)	226/459 (49.2)	231/464 (49.8)
Male sex, *n*/*N* (%)	229/459 (49.9)	257/463 (55.5)
Missing	0	1
Median (IQR) Infant age at randomisation in days	4 (3 to 6)	4 (3 to 6)
Birth weight < 10th centile for gestational age, *n*/*N* (%)	106/459 (23.1)	77/463 (16.6)
Missing	0	1
Median (IQR) Gestation at delivery (weeks)	29 (27 to 31)	29 (27 to 30)
Mean (SD) Birth weight (grammes)	1139.5 (331.8)	1131.8 (319.4)
Infant heart rate > 100 bpm at 5 mins, *n*/*N* (%)	421/455 (92.5)	418/456 (91.7)
Missing	4	8
Mean (SD) Infant temperature on admission (°C)	36.8 (0.7)	36.8 (0.8)
Missing	4	2
Mean (SD) Infant worst base excess within first 24 h of birth	− 6.0 (3.9)	− 6.1 (4.1)
Missing	8	10
Infant ventilated via endotracheal tube at randomisation, *n*/*N* (%)	90/458 (19.7)	107/463 (23.1)
Missing	1	1
Infant had absent or reversed end diastolic flow, *n*/*N* (%)	76/452 (16.8)	70/455 (15.4)
Missing	7	9
Mean (SD) Mother’s age at randomisation (years)	30.7 (5.8)	31.2 (6.5)
Multiple pregnancy^a,b^, *n*/*N* (%)	144/459 (31.4)	139/464 (30.0)
Singles^c^	0	1
Twins^d^	136	116
Triplets^e^	8	22
Caesarean section delivery, *n*/*N* (%)	284/459 (61.9)	272/464 (58.6)
Membranes ruptured before labour, *n*/*N* (%)	167/453 (36.9)	159/460 (34.6)
Missing	6	4
Membranes ruptured > 24 h before delivery, *n*/*N* (%)	119/454 (26.2)	104/458 (22.7)
Missing	5	6

^a^Minimisation factor

^b^Sometimes, only one infant from a multiple pregnancy met the inclusion criteria and was recruited

^c^Number of babies from multiple pregnancies where the other foetuses were aborted, miscarried or stillborn

^d^Number of babies who were one of twins

^e^Number of babies who were one of triplets

Unless otherwise stated the table gives the percentages of infants with data in that group of the trial who had

(or whose mother had) the stated characteristic

IQR denotes 25th percentile to 75th percentile; SD denotes standard deviation; bpm, beats per minute

### Primary outcome

Three hundred and eighty-one (381) infants had a questionnaire returned in the group allocated ‘Before’ (out of 459, 83.0%), compared with 353 returned out of 464 (76.1%) in the group allocated ‘After’ (see Table 2). There was strong evidence that an unconditional £15 incentive in advance (‘Before’ group) led to a statistically significantly higher response rate compared to a conditional incentive, with an absolute difference of 6.8% (95% confidence interval [CI] 1.6% to 12.0%, *p* = 0.01), adjusted for stratification factors (trial allocation and single or multiple birth).

**Table 2:** Primary and secondary outcomes in the SWAT

	Before group (***n*** = 459)	After group (***n*** = 464)	Unadjusted effect measure (95% CI)^a^	***p*** value	Adjusted effect measure (95% CI)^ab^	***p*** value
Questionnaire received (at 2 years of age corrected for prematurity)^c^, *n*/*N* (%)	381/459 (83.0)	353/464 (76.1)	6.9 (1.7, 12.1)	0.009	6.8 (1.6, 12.0)	0.010
Method of completion:						
Paper questionnaire, *n*/*N* (%)	326/459 (71.0)	295/464 (63.6)		0.061^**f**^		
Online completion, *n*/*N* (%)	50/459 (10.9)	53/464 (11.4)				
Completion via telephone, *n*/*N* (%)	5/459 (1.1)	5/464 (1.1)				
Total cost of the strategy^d^ (£)						
Mean (SD)	17.97 (1.7)	15.00 (6.7)	2.97 (2.33, 3.60)	< 0.001	2.98 (2.34, 3.61)	< 0.001
Median (IQR)	18.22 (17.41 to 18.40)	18.08 (18.08 to 18.88)				
Increase in cost compared to prior to commencement of incentives study (£, 95% CI)	14.80 (14.68, 14.94)	11.84 (11.51, 12.12)				
Reminder required^e^, *n*/*N* (%)	248/456 (54.4)	264/452 (58.4)				
0	208/456 (45.6)	188/452 (41.6)				
1	104/456 (22.8)	95/452 (21.0)		0.027^g^		
2	43/456 (9.4)	31/452 (6.9)				
3	101/456 (22.1)	138/ 452 (30.5)				
Missing	3	12				

^a^Absolute difference in the proportion responding and mean difference for cost

^b^Adjusted for stratification factors: trial allocation and single or multiple birth

^c^Receipt of a completed or partially completed (defined as completion of the first three sections or more) questionnaire at the SIFT office (note that the denominator is the number of eligible infants, which takes into account multiple births)

^d^Costs included postage, receipt of material via Freepost envelope, envelopes, sticker sets, and value of gift vouchers but excluded costs of Freepost licence fee, printing, telephone calls, and trial staff time

^e^Reminder 1 sent by post; reminder 2 sent by post plus accompanying phone call; reminder 3 phone call only

^f^*p* value for chi-squared test

^g^*p* value for chi-squared test for trend (linear association)

SD denotes Standard deviation; Interquartile Range (IQR) denotes 25th to 75th percentiles

### Secondary outcomes

#### Method of completion

Three hundred and twenty-six (326) questionnaires were completed on paper in the group allocated ‘Before’ (out of 459, 71.0%), compared with 295 in the group allocated ‘After’ (out of 464, 63.6%) (see Table 2). Completion rates online were broadly similar, with 50 (out of 459) returned in the group allocated ‘Before’ and 53 (out of 464) returned in the group allocated ‘After’ (10.9% and 11.4% respectively). Five questionnaires were completed over the phone in each group (1.1%). The difference in response rates appears to be dominated by completion on paper.

#### Cost

The mean cost of the incentive strategy per infant was £17.97 (standard deviation [SD] 1.7) in the group allocated ‘Before’, and £15.00 (SD 6.7) in the group allocated ‘After’ (see Table 2). The mean cost of the unconditional incentive scheme was £21.65 per response, compared with £19.72 per response for the conditional incentive. The mean cost per 1% increase in response is £1.35 per infant in the group allocated ‘Before’, compared with £2.95 per infant in the group allocated ‘After’.

The additional cost of the incentives strategies per infant compared to that prior to the incentives study was £14.80 (‘Before’ group) and £11.84 (‘After’ group). Unsurprisingly, providing the incentive in advance was more costly; however, the mean difference in the cost per infant was only £2.99 (95% CI £2.33 to £3.61).

#### Number of reminders

There was evidence to suggest that fewer reminders were required in the group allocated ‘Before’ (54.4%) compared to the group allocated ‘After’ (58.4%) (see Table 2). Furthermore, a higher proportion reached the third (final) reminder stage in the group allocated ‘After’ (138 out of 452, 30.5%) compared with the group allocated ‘Before’ (101 out of 456, 22.1%).

### Pre-specified subgroup analysis

**Fig. 2:**
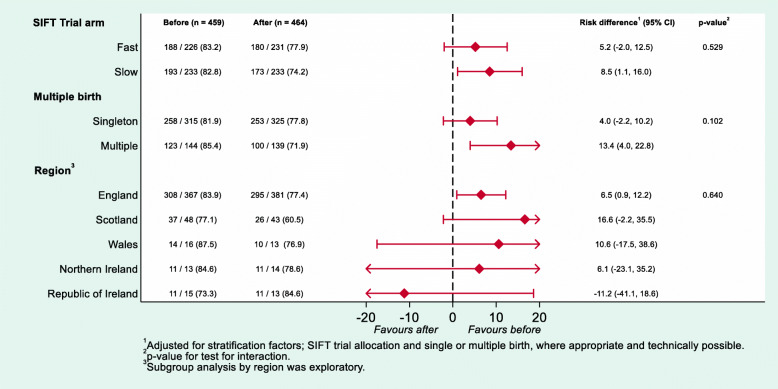
Subgroup analyses for response rate at 2 years

There was no evidence of a differential effect of the incentive strategy across the original allocation in SIFT (faster/slower feeds) or between single and multiple births. However, the response rate from parents of multiple births was higher in the group allocated ‘Before’ compared to the group allocated ‘After’ (see Fig. 2), but the test of interaction is not statistically significant, and the finding may be, in part, simply due to the increased amount (double or triple) received unconditionally.

In addition, in an exploratory subgroup analysis, we examined the response rate in terms of regional variation during the incentives study (see Fig. 2). However, since the vast majority of the infants were randomised in England, these results were difficult to interpret with some very wide confidence intervals. In the Republic of Ireland, the trend is reversed, while in Scotland response rates were lower in both groups.

### Pre-specified exploratory analysis

We examined the response rate prior to and during the incentives study overall i.e., irrespective of allocation (see Table 3). Again, not surprisingly, the response rate following the implementation of the vouchers was statistically significantly higher (79.5%, 95% CI 76.8% to 82.0%) compared to before implementation (72.1%, 95% CI 69.9% to 74.1%).

**Table 3:** Response rate at 2 years prior to and during the incentives study

Questionnaire received (at 2 years of age corrected for prematurity)	Prior to the incentives study (*n* = 1756)	95% confidence interval	During the incentives study (*n* = 923)	95% confidence interval
Questionnaire received (at 2 years of age corrected for prematurity), *n*/*N* (%)	1266/1756 (72.1)	(69.9, 74.1)	734/923 (79.5)	(76.8, 82.0)

## Discussion

In this SWAT, allocation to an unconditional incentive (‘Before’ group) produced a significantly more favourable response rate than allocation to a conditional incentive (‘After’ group), with an absolute increase of 6.8% (83.0% versus 76.1% respectively). This concurs with results from previous studies [3, 6]. The overall response rate was significantly higher after the SWAT was implemented, at 79.5% (95% CI 76.8% to 82.0) compared to 72.1% before (95% CI 69.9% to 74.1%).

The majority of questionnaires were returned by post and this method demonstrated the significant increase between groups. There was little difference between groups for the online or phone-supplied questionnaires, but there were relatively low numbers of responses overall via these two methods.

Both incentive methods are cost-effective in terms of increasing response rate. However, providing an unconditional incentive comes with an additional cost, in this case almost £3 per infant. The group allocated ‘Before’ led to an absolute increase in response rate of 11%, at an average additional cost of £14.80 per infant. The group allocated ‘After’ gave an absolute increase in response rate of 4%, at an average additional cost of £11.84 per infant. So, while the unconditional incentive is more expensive, it represents good value for money with a cost of £1.35 per infant for every 1% increase in response rate. This contrasts with the conditional incentives, which costs an additional £2.95 per infant for every 1% increase in response rate.

There was no evidence of a differential response rate between trial allocation or between single and multiple births. However, there was a greater response from parents of multiples on the group allocated ‘Before’. There could be a cumulative factor involved here, as parents of multiples received greater in advance incentives (£30 for twins, £45 for triplets). However, this promising single result has to be interpreted in the context of three similar studies. The totality of evidence regarding the effect on retention of the addition of a monetary incentive (unconditional) versus the addition of a monetary reward (conditional) remains highly uncertain though, especially in the presence of significant heterogeneity [13].

### Strengths and limitations

This SWAT nested within a perinatal RCT contributes to the evidence base for the effectiveness of incentives in this context. The study was run efficiently requiring minimal resources as it was integrated into the existing SIFT follow-up schedule. Ethics approval delays and logistics meant the SWAT study did not begin until February 2017, resulting in fewer participants still due to receive their questionnaire. Despite this, results showed a significant increase in the response rate in the group allocated an unconditional incentive (‘Before’ group).

This study is limited to a particular study population: parents of infants born extremely prematurely. They may be more likely to be interested in their infants’ progress. It is also limited to the follow-up juncture of 2 years. Given the scarcity of studies in the perinatal field, it is important to bear this in mind. Longer follow-up periods (for example at school age), and different modes of data collection and assessment (for example clinical assessment) and other incentive amounts may produce different results.

## Conclusion

This SWAT provides evidence that incentives can be a cost-effective strategy to maximise follow-up in perinatal RCTs. It also demonstrates that giving an incentive before parents complete their questionnaire is more effective than providing it after completion. While it is more expensive to use an unconditional incentive strategy, it pays dividends with a significantly higher response. This SWAT gives a clear indication of the potential benefit, which may be helpful to other trialists considering the same approach.

## Supplementary Information

**Additional file 1.**

## Data Availability

SIFT trial data are available upon reasonable request, subject to the NPEU Data Sharing Policy.

## Abbreviations

SIFT: Speed of Increasing Milk Feeds Trial
SWAT: Study Within a Trial
RCT: Randomised controlled trial
SD: Standard deviation
