# Supplementary material for: Evaluation of the effectiveness of an incentive strategy on the questionnaire response rate in parents of premature babies: a randomised controlled Study Within A Trial (SWAT) nested within SIFT
Source: Trials. 2021 Aug 21;22:554. doi: 10.1186/s13063-021-05515-y (PMC8379785; doi:10.1186/s13063-021-05515-y)
Supplement: Supplementary file 1 — Additional file 1. [file 13063_2021_5515_MOESM1_ESM.pdf]

[Name] [date]

[Address]

Dear [parent name]

Re: [child's name]

Thank you for agreeing to let [child's name] take part in the SIFT study at what must have been a very stressful time. We hope that you and [child's name] are feeling well. Now that [child's name] is two years of age we would like to find out about [his/her] health and development. We have enclosed a questionnaire for you to complete to tell us how [child's name] has been getting on since [s/he] was discharged from hospital.

This questionnaire is a really important part of the study. Your answers will help us find out which speed of feeding babies in the first few weeks of life is best for their overall development. This is the aim of the SIFT study - we are interested in hearing how [child's name] is doing.

There are three ways to give us this information:

1. Complete the questionnaire and post it back to me in the enclosed pre-paid envelope.

2. Complete the questionnaire online by typing the following link into your web browser:

   [unique URL]

   You will then be asked for an access code, which is: [access code]

   If you have provided us with your email address or mobile phone number you will be sent this link in an email or text message separately.

3. If you would prefer to answer the questions by telephone, please call [phone number]. I would be happy to call you back so you don't have to pay for the call.

Please be assured that any information you provide will be treated in the strictest confidence and will not be shared with anyone outside of the study. Please also note that this questionnaire will not replace any clinical follow-up at your local hospital.

We have enclosed some stickers for [child's name] to play with, and a £15 voucher as a thank you for taking the time.

If you have any queries or would like any help completing the questionnaire please contact me at sift@npeu.ox.ac.uk or by telephone on 01865 617919.

More information about the study can be found on the study website: www.npeu.ox.ac.uk/sift.

Thank you again for your support and for taking part in the SIFT Study; your contribution is greatly appreciated and will help shape the care of premature babies in the future.

With best wishes

Oliver Hewer
SIFT Trial Coordinator

UNIVERSITY OF OXFORD

National Perinatal Epidemiology Unit
Department of Public Health
University of Oxford, Old Road Campus, Headington, Oxford, OX3 7LF
T +44 (0)1865 289700 F +44 (0)1865 289701 www.npeu.ox.ac.uk

Speed of increasing milk feeds

[Name] [date]

[Address]

Dear [parent name]

Re: [child's name]

Thank you for agreeing to let [child's name] take part in the SIFT study at what must have been a very stressful time. We are so very sorry to hear that one of your babies died. We hope that you and [child's name] are feeling well. Now that [child's name] is two years of age we would like to find out about [his/her] health and development. We have enclosed a questionnaire for you to complete to tell us how [child's name] has been getting on since [s/he] was discharged from hospital.

This questionnaire is a really important part of the study. Your answers will help us find out which speed of feeding babies in the first few weeks of life is best for their overall development. This is the aim of the SIFT study - we are interested in hearing how [child's name] is doing.

There are three ways to give us this information:

1. Complete the questionnaire and post it back to me in the enclosed pre-paid envelope.
2. Complete the questionnaire online by typing the following link into your web browser:

   [unique URL]

   You will then be asked for an access code, which is: [access code]

   If you have provided us with your email address or mobile phone number you will be sent this link in an email or text message separately.
3. If you would prefer to answer the questions by telephone, please call [phone number]. I would be happy to call you back so you don't have to pay for the call.

Please be assured that any information you provide will be treated in the strictest confidence and will not be shared with anyone outside of the study. Please also note that this questionnaire will not replace any clinical follow-up at your local hospital.

We have enclosed some stickers for [child's name] to play with, and a £15 voucher as a thank you for taking the time.

If you have any queries or would like any help completing the questionnaire please contact me at sift@npeu.ox.ac.uk or by telephone on 01865 617919.

More information about the study can be found on the study website: www.npeu.ox.ac.uk/sift.

Thank you again for your support and for taking part in the SIFT Study; your contribution is greatly appreciated and will help shape the care of premature babies in the future.

With best wishes

Oliver Hewer
SIFT Trial Coordinator

UNIVERSITY OF OXFORD

National Perinatal Epidemiology Unit
Department of Public Health
University of Oxford, Old Road Campus, Headington, Oxford, OX3 7LF
T +44 (0)1865 289700 F +44 (0)1865 289701 www.npeu.ox.ac.uk

Speed of increasing milk feeds

[Name]

[Address]

Dear [parent name]

Re: [child's name]

About two weeks ago we sent a letter and £15 'thank you' voucher, asking you to complete a questionnaire as part of the SIFT study, which you kindly agreed to let your child to participate in soon after [he/she] was born. We appreciate that you are likely to be very busy, with little free time but would be very grateful if you would let us know how [child's name] has been getting on via this questionnaire. It is really important to us to know which speed of feeding babies is best for their overall development.

Please accept our apologies and disregard this reminder if you have recently sent us the completed questionnaire - we may not have received it yet.

There are three ways to give us this information:

1. Complete the questionnaire and post it back to me in the enclosed pre-paid envelope.

2. Complete the questionnaire online by typing the following link into your web browser:

   [unique URL]

   You will then be asked for an access code, which is: [access code]

   If you have provided us with your email address or mobile phone number you will be sent this link in an email or text message separately.

3. If you would prefer to answer the questions by telephone, please call [phone number]. I would be happy to call you back so you don't have to pay for the call.

Please be assured that any information you provide will be treated in the strictest confidence and will not be shared with anyone outside of the study. Please also note that this questionnaire will not replace any clinical follow-up at your local hospital.

If you have any queries or would like any help completing the questionnaire please contact me at sift@npeu.ox.ac.uk or by telephone on 01865 617919.

More information about the study can be found on the study website: www.npeu.ox.ac.uk/sift.

Thank you again for your support and for taking part in the SIFT Study; your contribution is greatly appreciated and will help shape the care of premature babies/infants in the future.

With best wishes

Oliver Hewer
SIFT Trial Coordinator

UNIVERSITY OF OXFORD

National Perinatal Epidemiology Unit
Department of Public Health
University of Oxford, Old Road Campus, Headington, Oxford, OX3 7LF
T +44 (0)1865 289700 F +44 (0)1865 289701 www.npeu.ox.ac.uk

[Name]

[Address]

Dear [parent name]

Re: [child's name]

About a month ago we sent a letter and £15 'thank you' voucher, asking you to complete a questionnaire as part of the SIFT study, which you kindly agreed to let your child to participate in soon after [he/she] was born. We appreciate that you are likely to be very busy, with little free time but would be very grateful if you would let us know how [child's name] has been getting on via this questionnaire. It is really important to us to know which speed of feeding babies is best for their overall development.

Please accept our apologies and disregard this reminder if you have recently sent us the completed questionnaire - we may not have received it yet.

There are three ways to give us this information:

1. Complete the questionnaire and post it back to me in the enclosed pre-paid envelope.

2. Complete the questionnaire online by typing the following link into your web browser:

   [unique URL]

   You will then be asked for an access code, which is: [access code]

   If you have provided us with your email address or mobile phone number you will be sent this link in an email or text message separately.

3. If you would prefer to answer the questions by telephone, please call [phone number]. I would be happy to call you back so you don't have to pay for the call.

Please be assured that any information you provide will be treated in the strictest confidence and will not be shared with anyone outside of the study. Please also note that this questionnaire will not replace any clinical follow-up at your local hospital.

If you have any queries or would like any help completing the questionnaire please contact me at sift@npeu.ox.ac.uk or by telephone on 01865 617919.

More information about the study can be found on the study website: www.npeu.ox.ac.uk/sift.

Thank you again for your support and for taking part in the SIFT Study; your contribution is greatly appreciated and will help shape the care of premature babies in the future.

With best wishes

Oliver Hewer
SIFT Trial Coordinator

National Perinatal Epidemiology Unit
Department of Public Health
University of Oxford, Old Road Campus, Headington, Oxford, OX3 7LF
T +44 (0)1865 289700 F +44 (0)1865 289701 www.npeu.ox.ac.uk

Speed of increasing milk feeds

[Name] [date]

[Address]

Dear [parent name]

Re: [child's name]

Thank you for agreeing to let [child's name] take part in the SIFT study at what must have been a very stressful time. We hope that you and [child's name] are feeling well. Now that [child's name] is two years of age we would like to find out about [his/her] health and development. We have enclosed a questionnaire for you to complete to tell us how [child's name] has been getting on since [s/he] was discharged from hospital.

This questionnaire is a really important part of the study. Your answers will help us find out which speed of feeding babies in the first few weeks of life is best for their overall development. This is the aim of the SIFT study - we are interested in hearing how [child's name] is doing.

There are three ways to give us this information:

1. Complete the questionnaire and post it back to me in the enclosed pre-paid envelope.

2. Complete the questionnaire online by typing the following link into your web browser:

   [unique URL]

   You will then be asked for an access code, which is: [access code]

   If you have provided us with your email address or mobile phone number you will be sent this link in an email or text message separately.

3. If you would prefer to answer the questions by telephone, please call [phone number]. I would be happy to call you back so you don't have to pay for the call.

Please be assured that any information you provide will be treated in the strictest confidence and will not be shared with anyone outside of the study. Please also note that this questionnaire will not replace any clinical follow-up at your local hospital.

We have enclosed some stickers for [child's name] to play with.

We will send you a £15 voucher as a thank you for taking the time, when we have received the completed questionnaire.

If you have any queries or would like any help completing the questionnaire please contact me at sift@npeu.ox.ac.uk or by telephone on 01865 617919.

More information about the study can be found on the study website: www.npeu.ox.ac.uk/sift.

Thank you again for your support and for taking part in the SIFT Study; your contribution is greatly appreciated and will help shape the care of premature babies in the future.

With best wishes

Oliver Hewer
SIFT Trial Coordinator

UNIVERSITY OF OXFORD

National Perinatal Epidemiology Unit
Department of Public Health
University of Oxford, Old Road Campus, Headington, Oxford, OX3 7LF
T +44 (0)1865 289700 F +44 (0)1865 289701 www.npeu.ox.ac.uk

[Name] [date]

[Address]

Dear [parent name]

Re: [child's name]

Thank you for agreeing to let [child's name] take part in the SIFT study at what must have been a very stressful time. We are so very sorry to hear that one of your babies died. We hope that you and [child's name] are feeling well. Now that [child's name] is two years of age we would like to find out about [his/her] health and development. We have enclosed a questionnaire for you to complete to tell us how [child's name] has been getting on since [s/he] was discharged from hospital.

This questionnaire is a really important part of the study. Your answers will help us find out which speed of feeding babies in the first few weeks of life is best for their overall development. This is the aim of the SIFT study - we are interested in hearing how [child's name] is doing.

There are three ways to give us this information:

1. Complete the questionnaire and post it back to me in the enclosed pre-paid envelope.

2. Complete the questionnaire online by typing the following link into your web browser:

   [unique URL]

   You will then be asked for an access code, which is: [access code]

   If you have provided us with your email address or mobile phone number you will be sent this link in an email or text message separately.

3. If you would prefer to answer the questions by telephone, please call [phone number]. I would be happy to call you back so you don't have to pay for the call.

Please be assured that any information you provide will be treated in the strictest confidence and will not be shared with anyone outside of the study. Please also note that this questionnaire will not replace any clinical follow-up at your local hospital.

We have enclosed some stickers for [child's name] to play with.

We will send you a £15 voucher as a thank you for taking the time, when we have received the completed questionnaire.

If you have any queries or would like any help completing the questionnaire please contact me at sift@npeu.ox.ac.uk or by telephone on 01865 617919.

More information about the study can be found on the study website: www.npeu.ox.ac.uk/sift.

Thank you again for your support and for taking part in the SIFT Study; your contribution is greatly appreciated and will help shape the care of premature babies in the future.

With best wishes

Oliver Hewer
SIFT Trial Coordinator

UNIVERSITY OF OXFORD

National Perinatal Epidemiology Unit
Department of Public Health
University of Oxford, Old Road Campus, Headington, Oxford, OX3 7LF
T +44 (0)1865 289700 F +44 (0)1865 289701 www.npeu.ox.ac.uk

Speed of increasing milk feeds

[Name]

[Address]

Dear [parent name]

Re: [child's name]

About two weeks ago we sent a letter asking you to complete a questionnaire as part of the SIFT study, which you kindly agreed to let your child to participate in soon after [he/she] was born. We appreciate that you are likely to be very busy, with little free time but would be very grateful if you would let us know how [child's name] has been getting on via this questionnaire. It is really important to us to know which speed of feeding babies is best for their overall development.

Please accept our apologies and disregard this reminder if you have recently sent us the completed questionnaire - we may not have received it yet.

You will still be eligible to receive a £15 voucher as a thank you for taking the time when we receive the completed questionnaire.

There are three ways to give us this information:

1. Complete the questionnaire and post it back to me in the enclosed pre-paid envelope.

2. Complete the questionnaire online by typing the following link into your web browser:

   [unique URL]

   You will then be asked for an access code, which is: [access code]

   If you have provided us with your email address or mobile phone number you will be sent this link in an email or text message separately.

3. If you would prefer to answer the questions by telephone, please call [phone number]. I would be happy to call you back so you don't have to pay for the call.

Please be assured that any information you provide will be treated in the strictest confidence and will not be shared with anyone outside of the study. Please also note that this questionnaire will not replace any clinical follow-up at your local hospital.

If you have any queries or would like any help completing the questionnaire please contact me at sift@npeu.ox.ac.uk or by telephone on 01865 617919.

More information about the study can be found on the study website: www.npeu.ox.ac.uk/sift.

Thank you again for your support and for taking part in the SIFT Study; your contribution is greatly appreciated and will help shape the care of premature babies/infants in the future.

With best wishes

Oliver Hewer
SIFT Trial Coordinator

UNIVERSITY OF OXFORD

National Perinatal Epidemiology Unit
Department of Public Health
University of Oxford, Old Road Campus, Headington, Oxford, OX3 7LF
T +44 (0)1865 289700 F +44 (0)1865 289701 www.npeu.ox.ac.uk

Speed of increasing milk feeds

[Name]

[Address]

Dear [parent name]

Re: [child's name]

About a month ago we sent a letter asking you to complete a questionnaire as part of the SIFT study, which you kindly agreed to let your child to participate in soon after [he/she] was born. We appreciate that you are likely to be very busy, with little free time but would be very grateful if you would let us know how [child's name] has been getting on via this questionnaire. It is really important to us to know which speed of feeding babies is best for their overall development.

Please accept our apologies and disregard this reminder if you have recently sent us the completed questionnaire - we may not have received it yet.

You will still be eligible to receive a £15 voucher as a thank you for taking the time when we receive the completed questionnaire.

There are three ways to give us this information:

1. Complete the questionnaire and post it back to me in the enclosed pre-paid envelope.

2. Complete the questionnaire online by typing the following link into your web browser:

   [unique URL]

   You will then be asked for an access code, which is: [access code]

   If you have provided us with your email address or mobile phone number you will be sent this link in an email or text message separately.

3. If you would prefer to answer the questions by telephone, please call [phone number]. I would be happy to call you back so you don't have to pay for the call.

Please be assured that any information you provide will be treated in the strictest confidence and will not be shared with anyone outside of the study. Please also note that this questionnaire will not replace any clinical follow-up at your local hospital.

If you have any queries or would like any help completing the questionnaire please contact me at sift@npeu.ox.ac.uk or by telephone on 01865 617919.

More information about the study can be found on the study website: www.npeu.ox.ac.uk/sift.

Thank you again for your support and for taking part in the SIFT Study; your contribution is greatly appreciated and will help shape the care of premature babies in the future.

With best wishes

Oliver Hewer
SIFT Trial Coordinator

UNIVERSITY OF OXFORD

National Perinatal Epidemiology Unit
Department of Public Health
University of Oxford, Old Road Campus, Headington, Oxford, OX3 7LF
T +44 (0)1865 289700 F +44 (0)1865 289701 www.npeu.ox.ac.uk
